# Area Dominates Edge in Pointillistic Colour

**DOI:** 10.1177/2041669518788582

**Published:** 2018-08-14

**Authors:** Jan Koenderink, Andrea van Doorn, Karl Gegenfurtner

**Affiliations:** Justus-Liebig Universität Giessen, Germany; University of Leuven (KU Leuven), Belgium; Utrecht University, the Netherlands; Justus-Liebig Universität Giessen, Germany; Utrecht University, the Netherlands; Justus-Liebig Universität Giessen, Germany

**Keywords:** colour, transitions, edges, macchie, textures, Pointillism

## Abstract

In Pointillism and Divisionism, artists moved from tonal to chromatic palettes, as Impressionism did before them, and relied on what is often called optical mixture instead of stirring paints together. The so-called optical mixture is actually not an optical mixture, but a mental blend, because the texture of the paint marks is used as a means to stress the picture plane. The touches are intended to remain separately visible. These techniques require novel methods of colour description that have to depart from standard colorimetric conventions. We investigate the distinctiveness of transitions between regions as defined through such artistic techniques. We find that the pointillist edges are not primarily defined by luminance contrast but are achieved in almost purely chromatic ways. A very simple rule suffices to predict transition distinctiveness for pairs of cardinal colours (yellow, green, cyan, blue, magenta, and red); it is simply distance along the colour circle or in the RGB cube. Distinctiveness of partition depends mainly on the colours of the regions, not the sharpness of the transition.

## Introduction

Pointillism and Divisionism (or Chromoluminarism) are artistic techniques that were widely explored at the end of the 19th century (Blanc, 1891; Clement & Houzé, 1999; Gage, 1987; Greene, 2007; Homer, 1964; Lee, 1987; Rewald, 1986; Signac, 1899).^1^ One often speaks of Neo-Impressionism (Clement & Houzé, 1999), perhaps an unfortunate term because of the fundamental artistic convictions involved. From the perspective of vision research, there are a variety of mutually distinct issues involved, whereas the effects obtained by the artists are not readily located in the mainstream vision research understanding. The bewildering variety of approaches explored by the artists and the huge gap between generic stimulus configurations used in vision science and those that excited the artists at the time are probably reasons that science has had little more than trivialities to add to the artists' empirical achievements.

**Figure 1.**
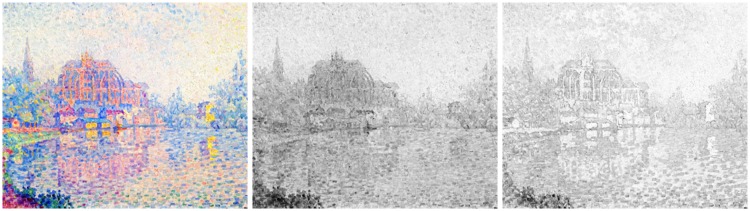
Paul Signac (1863–1935), Auxerre, *La Rivière* (1902–1903). Oil on canvas, height: 55.5 × 46.5 cm, private collection (image in the public domain). At left, a reproduction in colour; at centre and right, two attempts at monochromatic rendering. At the centre, the straight luminance image; at right, an interpretation using the “max rule” (see later); neither is particularly successful in capturing the spirit of the chromatic version. Alternative translations from hues to tones can do much better (see later) but necessarily involve idiosyncratic interpretation.

In this study, we explore two technically related aspects that appear of generic importance to the general area of pointillist and divisionist techniques. One involves the nature of *edges*, *boundaries*, or *transitions*, or whatever one choses to call them (Koenderink, van Doorn, Pinna, & Wagemans, 2016), the other the difference between mainly “chromatic” versus mainly “tonal” distinctions. A primarily chromatic work is almost impossible to reproduce in a monochrome rendering (see Figure 1).

Although these are indeed important issues, they do by no means exhaust the pointillist–divisionist toolbox. The sheer variety of effects that play in these art forms defeats simplistic, abstract scientific approaches.

This study is no more than an initial attempt to explore these matters. We state upfront that we cannot claim to do more than scratch the surface. Apart from its immediate application to the theory of the visual arts, this study is of interest to vision research per se because relatively little is known concerning the saliency of transitions between chromatically defined textures (Agostini & Galmont, 2000; Gegenfurtner & Kiper, 1992; Giesel, Hansen, & Gegenfurtner, 2009; Goda & Fujii, 2001; Hansen, Giesel, & Gegenfurtner, 2008; Ishihara, 1917; Li & Lennie, 1997; McIlhagga, Hine, Cole, & Snyder, 1990; Papathomas, 1997; Pessoa, Beck, & Mingolla, 1996; te Pas & Koenderink, 2004) as opposed to transitions between uniform (“flat”) patches of colour (Boynton, 1978; Boynton & Kaiser, 1968; Chevreul, 1860; Kaiser, 1971; Kaiser, Herzberg, & Boynton, 1971; Kaiser, Lee, Martini, & Valberg, 1990; Koenderink et al., 2016; Liebmann, 1927; Lindsey & Teller, 1989; von Helmholtz, 1867).

The dichotomy between textures and boundaries (or abutting patches) is not categorical; for instance, the studies using grating patterns occupy an intermediate position (Noorlander & Koenderink, 1983). However, as a coarse dichotomy, the distinction tends to be quite useful.

### Regions, Transitions, Boundaries, and Edges

Patches of a similar nature—tonally, chromatically, texturally, as the case may be—are major components of many paintings, technically denoted *macchie, macchia* being Italian for larger dabs of colour (Boime, 1993; Broude, 1987; Panconi, 1999).

Frequently, patches come with boundaries that naturally belong to the patch, but especially abutting patches often share a mutual area of transition. An instance of the former might be an occluding edge (or cutting edge), and an instance of the latter is the terminator of a body shadow. At an occluding edge, a foreground object occludes a background that runs on behind the object; thus, this hard edge belongs to the object; it is one-sided. At the edge of a body shadow, both regions belong to the same object, so they must naturally share a common boundary; this soft edge is two-sided (Koenderink et al., 2016).

**Figure 2.**
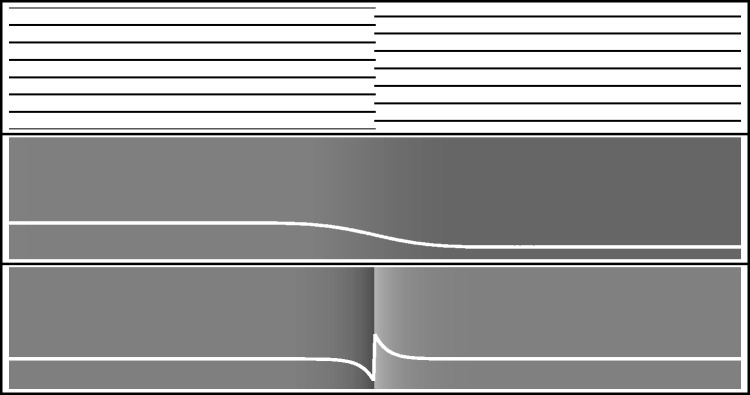
At top, two uniform areas of stripes abut in a nonexistent edge. At centre, two uniform, but mutually different areas are connected by way of a smooth transition; there is hardly a notion of an edge. The areas are perceived as similar. At bottom, two identical uniform areas are divided by a local edge. The areas are perceived as different.

Sharp transitions tend to be of a linear nature (mainly extended in a single dimension) and are often named (hard) edges. Transitions are often thought to *divide*. However, for compositional reasons in painting, they often need to *unite*, that is, glue together distinct regions (Koenderink, van Doorn, & Pinna, 2015; Koenderink et al., 2016). Thus, painting involves a spectrum of edges from hard to soft or even lost (lost edges are artistically important because they create *passages* that are most helpful in composition. Moreover, the challenge they offer to the eye—Gombrich's beholder's share—is considered to be an important artistic tool; Gombrich, 1960). Notice that such lost (nonexistent) edges are nevertheless phenomenologically present—think of the Kanizsa triangle (Kanizsa, 1955)—just as an edge may divide or connect nondifferentiated regions—think of the Cornsweet effect (Cornsweet, 1970). Figure 2 has some examples in a nonpointillist, grey scale style.

Regions, transitions, boundaries, and edges are major raw materials of the painter. Any novel technique that allows one to control these entities implies a major step forward in the technique of the art. Pointillism provides a major instance of that.

In classical painting, predominantly flat areas like a blue sky are rendered as shallow gradients (Ruskin, 1857). They contain no points, for no part of the gradient contrasts with its immediate environment. In contradistinction, in Pointillism, the blue sky is composed of points (separately recognisable dots of paint). However, these have no individual pictorial meaning but are perceived as elements of a texturally uniform patch. The textural quality unites the patch, as effectively as a flat tone or colour would. The artistic advantage is that the sense of the picture plane is retained, thus avoiding the impression that the picture is a “window” or a “hole in the wall.” Such an impression was actually thought of as desirable in the early 19th century but was considered in the worst of taste at its conclusion.

In classical painting, edges are mainly controlled by blurring (the oil technique being eminently suitable), or, when the painter has to leave brushstrokes visible as in classical tempera painting, by some technique of cross-stitching. Pointillism yields a novel possibility in that its points—again small dots of paint that have no pictorial meaning as such—may be made to cross borders and invade the adjoining macchie. Such techniques had long been used by landscape painters, who introduced “sky-holes” in foliage and floating leaves in the adjoining air space (Ruskin, 1857). This unites what is (and should remain) distinct much more effectively than a transitional region of locally mixed colours would. A similar advantage results from applying only partly mixed paints with a palette knife.

In this study, we address the visual impact of such transitions between regions. These important entities have hardly been studied in vision science, which is primarily focussed on (hard) edges between flat areas of colour.

When visual awareness is primarily of a transition, we will use the term *(edge) salience*. If the awareness is primarily of different regions, we will use the term *(macchia) distinctiveness* (Boime, 1993).

Notice that one may have saliency without distinctiveness (as in the Cornsweet stimulus) and likewise distinctiveness without saliency as in the case of very soft edges (as often used in vision science to demonstrate effects of local adaptation).

Thus, salience and distinctiveness are distinct categories.

### Chromatic Versus Tonal Contrasts

Up to the advent of French Impressionism, most painting depended upon tonal values. This implies a rather dark overall tonality and generally subdued chromatism. In contradistinction, in the novel styles, tonal contrasts were suppressed; the overall tone became lighter and chromatic modulations took over (Denvir, 1990; Eisenman, 2011; Moffett, 1986; Rewald, 1973).

In Divisionism, one often used a very limited palette of cardinal colours. (The painter's primary colours yellow, red, and blue, and the secondary colours orange, green, and purple, but for our—slightly different—definition, see later.) Mixtures could be left to the eye, or became “optical.” The term *optical mixture* easily leads one astray though. To be artistically effective, the mixture should be by the mind, not the light. That is to say, the chromatic dots should remain clearly visible, not be blurred or otherwise mixed.

For instance, Seurat's paintings (early Pointillism) look disappointingly drab and greyish from a distance, whereas the canvas comes to life when seen from close by. This is the reason why later artists often increased the sizes and spacings of their dots, or even spread them out, leaving the primed canvas between them visible.

An additional reason for the latter trend is the growing need towards the end of the 19th century to break free from the notion of a painting as a window (a view into another space through a virtual aperture) to that of a decorative flat object. In the former case, the picture surface is downplayed; in the latter, it is emphasised.

In vision science, such decorative surfaces are considered textures and have been mainly studied for their statistical spatial structure. The properties of mental chromatic mixtures have hardly been addressed, although their importance has often been acknowledged (Giesel et al., 2009; te Pas & Koenderink, 2004).

### Scope of This Study

In this study, we consider chromatic contrasts for hard versus soft transitions, and two kinds of colour gamuts. In all cases, the rendering is pointillistic, thus setting the stimuli apart from the vision science mainstream.

One of our main research questions is whether purely chromatic contrasts can hold their own against tonal (or luminance) contrasts. This is a major issue, since by the end of the 19th century artists aimed at uniform overall tonalities so as to stress the object character of the picture surface. Thus, they had a need for building contrasts in alternative ways.

From a scientific point of view, one wonders whether these chromatic contrasts were perhaps not really *luminance* contrasts (Koenderink, van Doorn, & Gegenfurtner, 2017a, 2017b). For the intrinsic luminances of the primary colours (RGB) are rather different, the ratio for our display being R:G:B = 21:62:17 (see Methods section), or anything close to 3:6:1 perhaps being most typical. However, there are reasons to doubt that luminance contrast would account for transition saliency, one reason being that monochrome renderings of chromatic (as opposed to tonal) paintings are generally disappointing (Lee, 1987), even in cases where the topic as such might well be conveyed in monochrome (example shown in Figure 1).

To study this, we collect a varied body of empirical data and attempt to account for it in terms of some simple models. Two of the models involve Commission Internationale de l'Éclairage (CIE) luminance, either directly or indirectly as in CIEDE2000 (Luo, Cui, & Rigg, 2001). The third model simply uses Euclidean distance in the display RGB cube for CIE D65 white and standard gamma 2.2. We are well aware that, from an “official” perspective, the latter choice is a no-go area, but in view of—perhaps surprising—previous results (Koenderink et al., 2017a, 2017b), we consider it anyway. After all, if really nonsense, that should immediately cause a clash with the data.

Another research question involves the nature of transitions between regions. Two opposing views would be to consider transitions primarily as hard edges versus some more gradual intermediary between two different regions. In the former case, the transition itself would be the important aspect; in the latter case, it would rather be the distinction between two mutually nonabutting regions. The transition either divides or unites, and the painting has a linear (pattern of edges) or a patchy (pattern of macchie) character.

Vision research predominantly considers hard edges—the bipartite aperture being preferred (Boynton, 1978; Boynton & Kaiser, 1968; Kaiser, 1971; Kaiser et al., 1971, 1990)—rather than patches—the simultaneous comparison of distinct apertures being rare. The reason is that in the case of vanishing contrasts, the former method leads to objective threshold measurements, whereas the latter retains a subjective character.

In this study, we consider supraliminal contrasts throughout. Our present methods are experimental phenomenology (Albertazzi, 2013) rather than psychophysics proper (thresholds or just noticeable differences).

This is appropriate enough for the present topic, for painting has nothing to do with either absolute or increment thresholds. In the arts, only phenomenology counts.^2^

## Methods

### Stimuli

The stimuli were designed so as to mimic pointillist/divisionist rendering in a generic way, mainly aimed at the later phase of these movements. We strive at stimulus structure that also has a generic relevance to vision science.

The dots are individually visible, the background at various places being visible in between. The colours are slight variations on the cardinal colours (Koenderink, 2010; Küppers, 1976; Schopenhauer, 1816; von Goethe, 1810): red, green, and blue (R, G, B, the primary colours) and turquoise (or cyan), purple (or magenta), and yellow (C, M, Y, the secondary colours). (Here, we use RGB colour cardinals, RGB and CMY, rather than the painter's YRB and OGP, as noticed earlier.)

Of course, one has that cyan is green and blue (C = G ∪ B), magenta is red and blue (M = R ∪ B), and yellow is red and green (Y = R ∪ G), where the “and” (or ∪ ) connective stands for “union,” or additive mixing. Blending of varicoloured texture relies not so much on optical factors (we neither blur the stimulus patterns nor view them from a distance), but rather the generalising capacity of visual awareness, sometimes referred to as assimilation in vision science. In this article, we refer to “blending” in that case, whereas we reserve “mixing” for colorimetric or optical operations.

We use a set of six fiducial hues, the periodic sequence YGCBMR (see Appendix A). This grain size is in the general ballpark of divisionist practice. For ease of reference, instead of specifying hue numerically, by cyclical index, we use capital letters (Y, C, B, M, R) or simply coloured patches to indicate axes ticks.

#### Spatial structure

**Figure 3.**
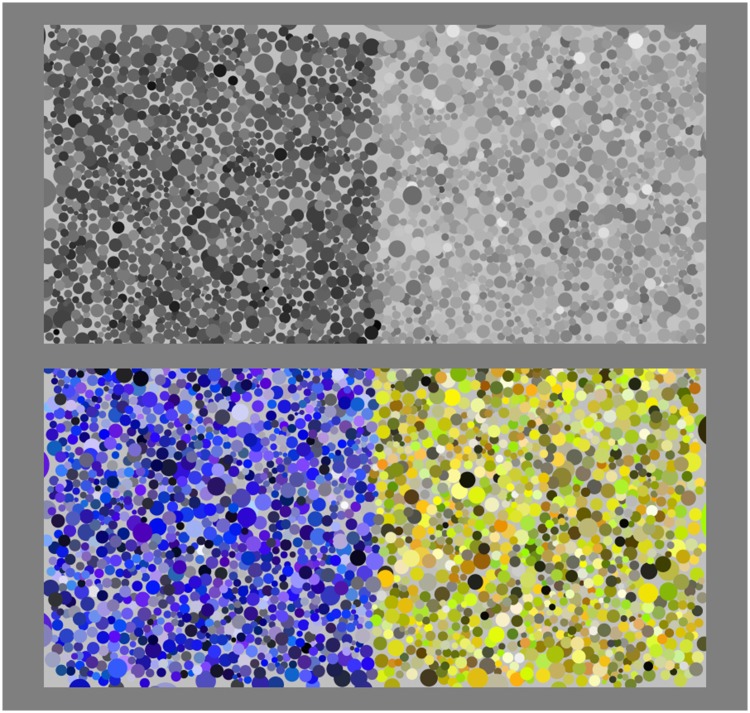
Example of the hard-edge stimulus. On top, the achromatic reference; at bottom, a polychromatic case. The structure of the colour gamuts and the relevance of the global layout are discussed later.

**Figure 4.**
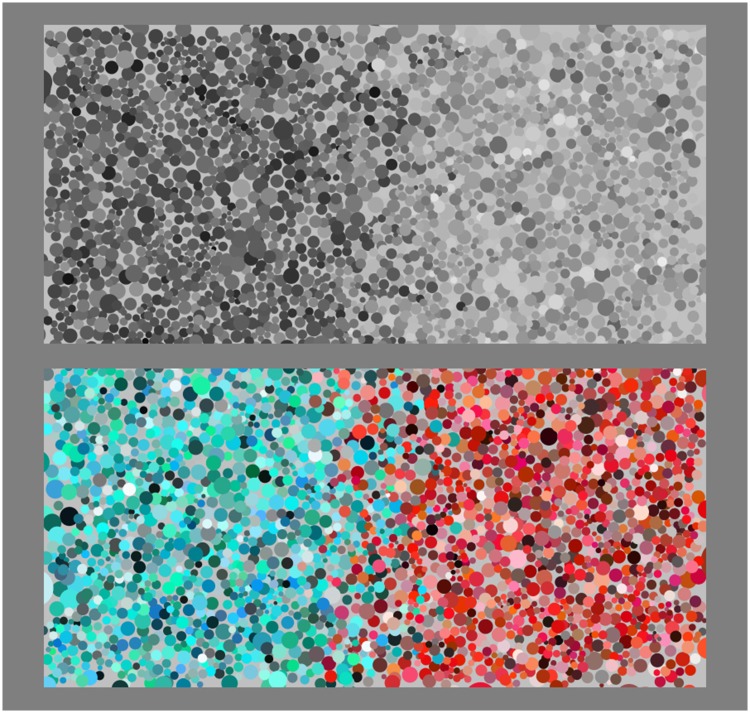
Example of the soft-edge stimulus. On top, the achromatic reference; at bottom, a polychromatic case. The structure of the colour gamuts and the relevance of the global layout are discussed later.

The stimuli are made up of two square patches, abutting at a common transitional strip of some width (Figures 3 and 4).

The pointillist structure is generated as follows: First, locations are defined in the union of the squares using a uniform random generator. For these points, a Voronoi tessellation (Aurenhammer, 1991) is prepared. For each (necessarily convex) face of the tessellation, we determine the barycentre and the shortest distance over all vertices from that centre. The dots are circular disks, centred at the barycentres, with radius taken as the shortest distance times a common factor. The common factor is taken such as to achieve a desired filling factor (fraction of background remaining uncovered).

Two types of transition are implemented, a relatively sharp and a relatively soft one (examples shown in Figures 3 and 4; various details are explained later). For the sharp transition, referred to as a *hard edge*, a dot is classified left or right according to the location of its centre. For the soft transition, referred to as a *soft edge*, a boundary width is defined in which the probability of being designated left or right varies linearly over the boundary width.

The following are some numerical data (notice that, e.g., Figure 3 shows four regions):

**Table:** 

Number of random points	5000 (ca. 71 × 71 dots per region)
Each region size	400 × 400 pixels
Frame rate	40 Hz
Transition width	40 pixels

#### Chromatic structure

We use three distinct types of colour gamuts (Bouma, 1946; Koenderink, 2010; Ostwald, 1917), namely,

Achromatic: These are greys on a linear scale from black to white;

Monochromatic: These are tints and shades of a single hue. They are naturally parameterised using the colour content, white and black content;

Polychromatic: These are shades and tints of a set of distinct hues. They are naturally parameterised by hue, colour content, white and black content.

These gamuts have different functions in the experiment. Their structures are defined later.

This implementation is at least reminiscent of gamuts seen in many later pointillist/divisionist works.

#### Achromatic gamut

**Figure 5.**
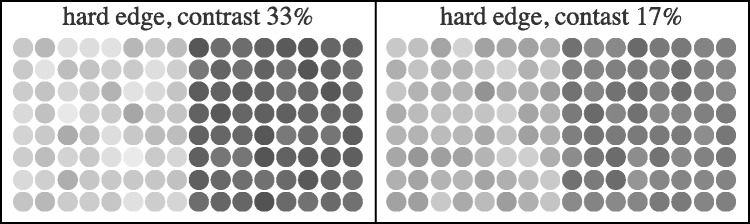
Schematic examples of hard achromatic edges. Notice that the left and right regions are both nonuniform; yet, the contrast of the overall pattern is well defined.

In the achromatic case, the dots of the texture are assigned some average intensity on which a random perturbation intensity on a per dot basis is superimposed. This dither of tone was determined by a normal distribution of fixed variance (schematic examples shown in Figure 5). The contrast of the perturbation amounted to a (indeed, fixed) standard deviation of 20% of the full black–white scale.

In the case of our bipartite stimuli, the contrast, which is the variable used to quantify responses, is defined as the Michelson contrast (Michelson, 1927) between the averages at both sides of the transition, thus,

$$c=|\frac{〈I_{1}〉-〈I_{2}〉}{〈I_{1}〉+〈I_{2}〉}|,$$

where $〈I_{1}〉,〈I_{2}〉$ denote the two averages.

#### Monochromatic gamuts

In the monochromatic case, the hue is fixed to one of the cardinal colours YGCBMR (Quiller, 1989). The colour *Q* of a dot in the texture is a mixture of the full colour *F*, white *W*, and black *K* with coefficients {*c, w, k*} satisfying c+w+k=1 (Koenderink, 2010; Ostwald, 1917), thus,

Q=cF∪wW∪kK.

(Where the last factor may be omitted, making no effective difference. It is formally added to remind one that this description relies on the existence of a white reference. Without that the notion of black content cannot even be consistently defined. See also Appendix A.)

In the texture, we assign the coefficients randomly. The random generator is somewhat skewed so as to favour saturated colours over weak tints and shades. This is done by generating triplets of numbers that are uniformly distributed over the unit interval and then multiplying the first number with a skew factor, after which the triplet is normalised by dividing by their common sum. In our stimuli, we use a skew factor of four. From an informal study, we glean that such a distribution mimics the practice often, though of course only approximately, seen in pointillist paintings. (There appears to be no systematic science on the topic.)

**Figure 6.**
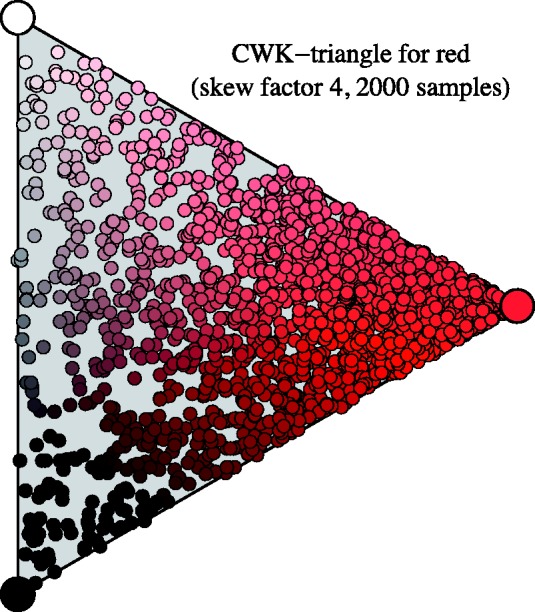
Monochromatic colour samples in the CWK (Ostwald) triangle (see Appendix A). Notice that the distribution is skewed so as to favour saturated colours over tints and shades of the hue.

In Figure 6, we show some samples for red.

#### Polychromatic gamuts

**Figure 7.**
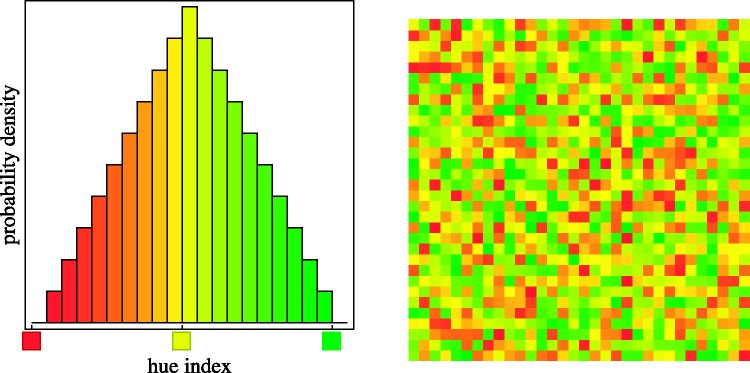
Polychromatic hue samples from a triangular distribution (see Appendix A). In this example, the average hue is yellow, and outliers range from red to green. The painter would speak of analogous or related colours. (Probability density on a linear scale, hue index defined in Appendix A.)

In this case, the hue is dithered through a triangular probability density function, centred at the mean and extending one step along the colour circle to each side (see Appendix A). (Thus, for yellow, the range runs from red to green, strongly favouring yellow; see Figure 7.) The spread is one step on the six-step colour circle, as artist often call it a palette of analogous colours (Quiller, 1989). In Figure 7, we illustrate such a distribution of hues, centred on yellow.

In the final polychromatic gamuts, we additionally applied the skew dithering in the CWK triangle (see Appendix A) as discussed in the section on monochromatic gamuts.

### Setup and Observers

#### Implementation

Stimuli were presented on the LCD screen of an Apple MacBook Pro 15″ (mid-2007 model). The colorimetric definitions of the primary cardinal colours are known from a photospectrometric calibration (Foley, van Dam, Feiner, & Hughes, 1990). The screen was viewed in a darkened room from a distance of 57 cm, using binocular vision. The screen subtended 32 ° × 20 °. Each square subtended a visual angle of 9 ° × 9 °.

The display was linearised using Bergdesign SuperCal 1.2.4. Measurements were done using gamma 2.2 (Poynton, 1993), results converted to luminance.

Photometric data on the display are (as measured with an X-Rite ColorMunki photospectrometer) as follows:

**Table:** 

Red	x = 0.5995	y = 0.3406	L = 68.9
Green	x = 0.3259	y = 0.5723	L = 197.4
Blue	x = 0.1534	y = 0.1346	L = 53.2

#### Observers

Five observers completed all sessions. This was considered amply sufficient in view of the excellent concordance of their results and the minor spreads in the observations (see Results section).

Three of the observers were fully naive to the task and had none or little and unrelated experience with vision research. Two of the observers were highly experienced in numerous visual tasks (the authors), although also new to the present one.

All observers were normal trichromats as determined by the conventional Ishihara test (Ishihara, 1917).

Genders include males (2) and females (3); ages range from 20s to 70s.

### Experimental Phenomenology

In this type of research, one needs to rely on eye-measure, or experimental phenomenology (Albertazzi, 2013), as the research questions cannot possibly be addressed through fully objective psychophysical techniques.

We displayed achromatic and chromatic stimuli simultaneously and gave observers control over the contrast of the achromatic one.

The task was to set this contrast such that the pair of squares in achromatic and the pair in colour (monochromatic or polychromatic as the case may be) appeared “mutually equally distinct.” Thus, the response is a kind of saliency measure. Of course, we cannot know whether the observers experience primarily edge salience or region distinctiveness.

**Figure 8.**
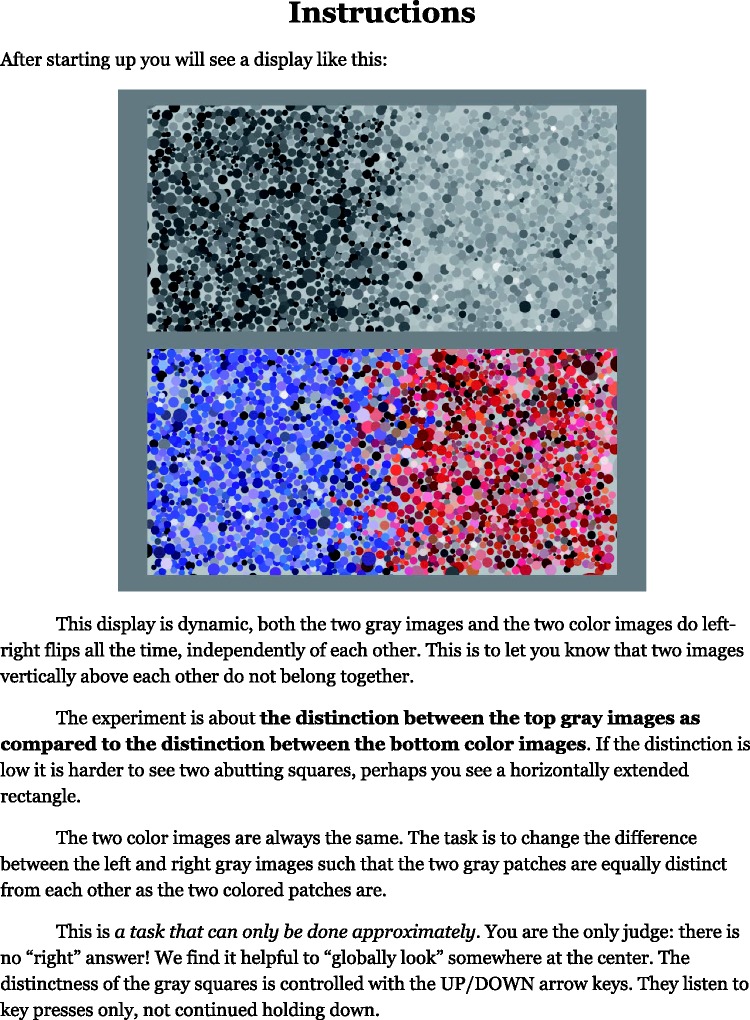
The instruction sheet used in the experiment.

The formal task was provided as a printed page to be read by each participant before the experiment. The wording carefully avoided technical terms such as “edge,” “luminance,” “contrast,” and so forth (see Figure 8).

“Mutually equally distinct” is what is important pictorially. No participant had any problem with this; the task appeared natural to them, although it was clear that it had to be done on the guts, using pure eye-measure. It was pointed out in the formal instruction that *the observer is always right, and there is no “correct” answer*.

Achromatic or tonal contrast was changed in 10% steps, starting from a random level. Participants controlled it by way of the up/down arrow keys on the keyboard.

Notice that this is not a psychophysical measurement of contrast. The contrast value set by the observer is interpreted as a subjective evaluation of the mutual dissimilarity of the left and right patches. We refer to it as the distinctiveness of the bipartition.

**Figure 9.**
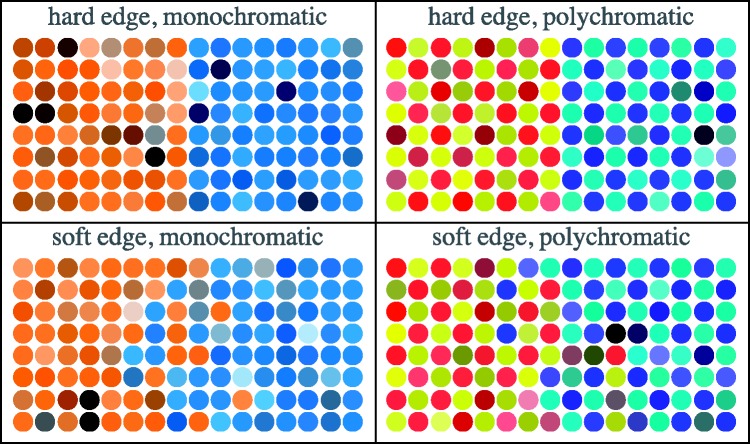
A schematic overview of the stimuli used in each condition. (Impressions of the actual screen images were presented in Figures 3 and 4.) The columns show monochromatic colour gamuts at left and polychromatic colour gamuts at right. The rows show hard edges at top and soft edges at bottom. In all these cases, we test all pairs of cardinal hues, thus YG, YC, YB, YM, YR, GC, GB, GM, GR, CB, CM, CR, BM, BR, and MR. The reader might find it useful to keep this schematic overview in mind. In referring to any case, we will specify the colour pair (like YR, say), the nature of the transition (“hard” or “soft”), and the nature of the colour gamut (“monochromatic” or “polychromatic”). In all cases, the response will be the Michelson contrast of the matching (qua “distinctiveness of the bipartition”) achromatic image.

The achromatic rendering was always presented on top. The right and left patches for both achromatic and chromatic squares were randomly swapped at quarter second intervals, independently. Thus, there was no fixed vertical relation between the squares. This implies that one cannot decide whether “yellow is brighter than blue” or vice versa, but only that the yellow–blue distinction is of a certain visual importance relative to (say) the cyan–red distinction. In a session, participants visited all pairs of distinct cardinal colours in random order. They completed four different conditions (Figure 9) in a single session of less than an hour (closer to half an hour, see later), in randomly chosen order.

Response times were left free but were recorded. Response times and contrast settings were written out as the data files on which the analysis is based.

### Stimuli Used in the Experiments

The stimulus categories are summarised (very schematically) in Figure 9.

Because this experiment is quite complicated with regard to the stimulus choices, the reader might find it useful to refer to Figure 9 in case of doubt or confusion. The terms hard edge and soft edge will be used to denote the spatial configuration, and the terms monochromatic and polychromatic will be used to denote the chromatic configuration.

The term *achromatic* will hardly be used; instead, the response will be specified numerically, as the Michelson contrast of the matching achromatic pattern.

“Matching” will denote equal distinctiveness of bipartition. This may equally well relate to edge saliency or to the distinctiveness of left/right regions. We do not (and indeed cannot) know. It may even be different for different observers. Most likely, observers themselves are not aware of the difference.

## Experiments

Median response times varied from 7 s to 12 s, several seconds being needed to arrive at the desired level starting from the random initial value. Observers typically went up and down a few times, before setting on their final level. There were no significant differences between the four conditions.

Observers agree quite well in their settings, as judged by Kendall's tau rank correlation. For the pooled data (240 responses per observer), the median rank order correlations is .74 with very little spread. It is more interesting to look at a breakdown in categories (omitted entries are not significant at the 5% level); see Table 1.

**Table 1.** A Breakdown in Categories of Kendall's Tau Rank Correlations of Pairwise Observer Responses.

	AL	AD	JK	KD	ZM
Soft monochromatic					
AL	1.00	.28	.41	.45	–
AD	.28	1.00	.41	.37	.43
JK	.41	.41	1.00	.35	.56
KD	.45	.37	.35	1.00	.37
ZM	–	.43	.56	.37	1.00
Soft polychromatic					
AL	1.00	.60	.58	.52	.35
AD	.60	1.00	.37	.66	.52
JK	.58	.37	1.00	.49	.28
KD	.52	.66	.49	1.00	.45
ZM	.35	.52	.28	.45	1.00
Hard monochromatic					
AL	1.00	.58	–	.49	.45
AD	.58	1.00	.37	.60	.60
JK	–	.37	1.00	–	.35
KD	.49	.60	–	1.00	.43
ZM	.45	.60	.35	.43	1.00
Hard polychromatic					
AL	1.00	.50	.43	.35	.54
AD	.50	1.00	.47	.66	.81
JK	.43	.47	1.00	.50	.35
KD	.35	.66	.50	1.00	.54
ZM	.54	.81	.35	.54	1.00

**Figure 10.**
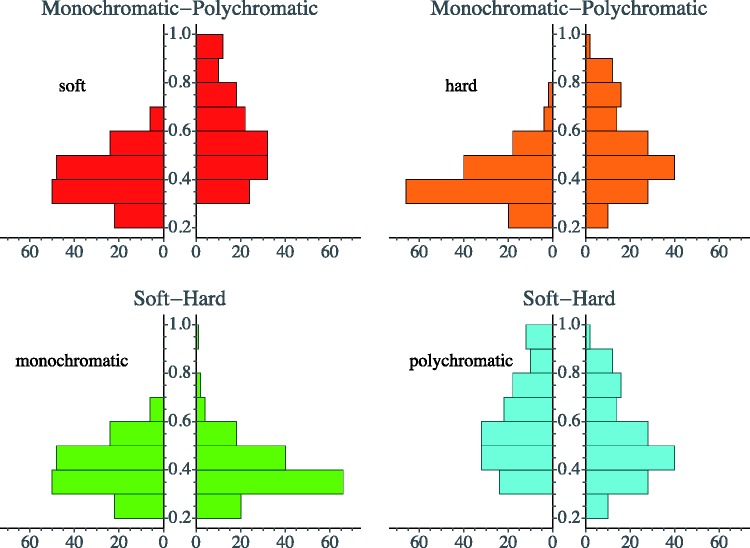
Paired histograms of the responses over all observers and colour pairs, differentiated with respect to the stimulus categories. Notice that the dichotomy monochromatic–polychromatic yields a large difference and that of soft–hard edge quality rather less so. (All counts marked on the same scale for convenient comparison.)

Contrasts are generally in the 36% to 55% range (interquartile range), with outliers in the 21% to 99% range (total range), the variation over the cardinal colour pairs perhaps being remarkable limited (see later). The distribution charts shown in Figure 10 show this global result.

**Figure 11.**
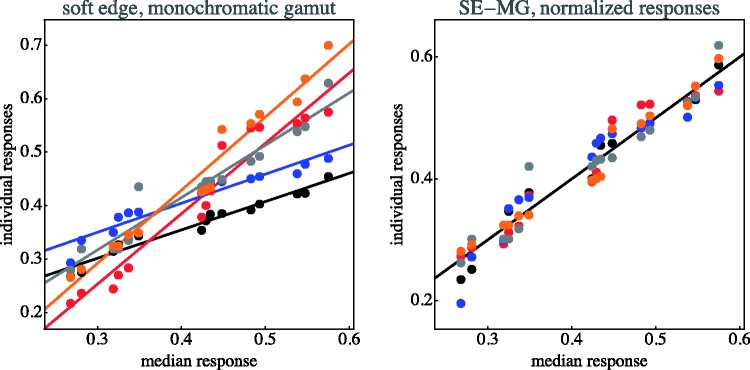
Distinctiveness responses are plotted against the median of the distinctiveness response over observers. Original data plotted at left; observers are distinguished by colour. The lines are fits for each observer. At right, the same data normalised with respect to idiosyncratic offset and slope of the individual observers. (SE–MG stands for “soft-edge, monochromatic gamut”)

The individual data items for each case are collected in Figures 11 through 14. This shows that—as indeed expected—observers have idiosyncratic offsets and gain factors, an aspect that is lost in the global view provided by Figure 10. In the remainder of our analysis, we use normalised data.

**Figure 12.**
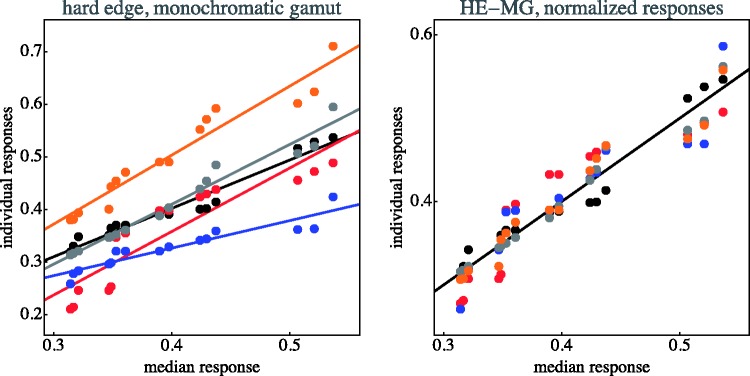
As Figure 11; here, HE–MG stands for “hard-edge, monochromatic gamut.”

**Figure 13.**
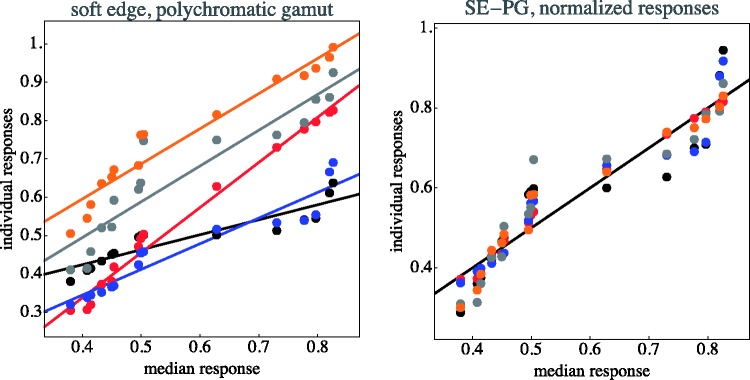
As Figure 11; here, SE–PG stands for “soft-edge, polychromatic gamut.”

**Figure 14.**
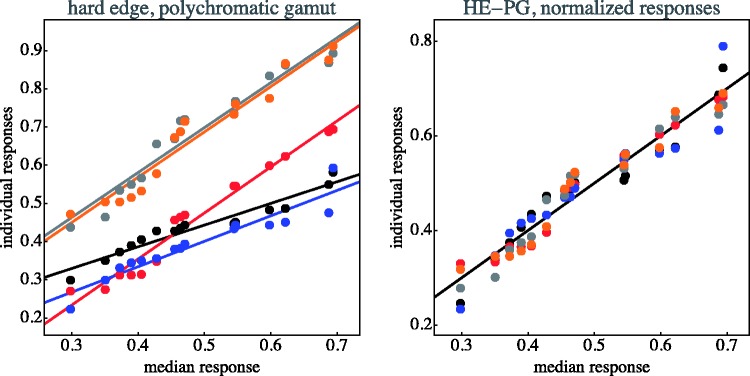
As Figure 11; here, HE–PG stands for “hard-edge, polychromatic gamut.”

The normalisation as illustrated in Figures 11 through 14 proves to be very effective. This is typical when observer results differ by some idiosyncratic parameter, which is apparently the case here. Omitting such a normalisation step would seriously misrepresent the results. A preliminary step like this in the analysis of multiobserver data is almost always called for in experimental phenomenology and is frequently of crucial importance (Koenderink, van Doorn, Kappers, & Todd, 2001).

The actual concordance—that is, with respect to the normalised data—between observers is much better than might naïvely have been expected on the basis of the uncorrected data. It suggests that participants are indeed doing a similar task, albeit with idiosyncratic traits that show up in the raw results, but are not really related to the actual task. On the basis of this result, we decided that the initial choice of five observers is to be considered sufficient.

### Overall Results

It is difficult to provide an intuitive overall view of all the data because of the many parameters involved. In Appendix C, we present bar plots of all data (medians and interquartile ranges over observers) as a function of stimulus category and colour pairs. Appendix B has an extensive explanation on the stimulus structure as relevant for the understanding of the results. We do not believe it to be necessary to go into that much detail in the main text. Here, we present a selection, yielding a more focussed view of the data.

**Figure 15.**
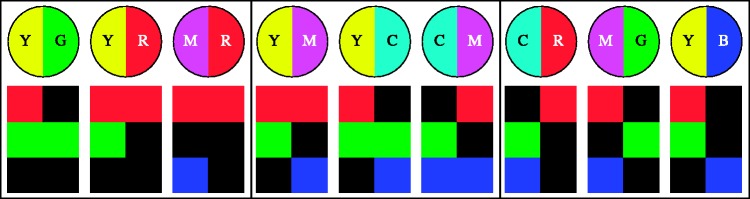
Some examples of chromatic transition structure. In each case, there is the chromatic transition shown in the bipartite disk at top; below it is an analysis in terms of the RGB colour channels. The RGB colour channels are the rows; the two columns represent the left and right regions of the transition. There are three groups of examples; at left, there is a transition in one, at centre in two, and at right in all three of the RGB colour channels. The first group illustrates the influence of the colour of the veil; the second group illustrates the effect of the absence or presence of a veil, whereas the third group illustrated the effect of the (relative) polarity of transitions in the individual RGB colour channels.

One may differentiate the data with respect to the bipartite differences in the three RGB colour channels (see Figure 15 and Appendix B). The partition may be in the red, the green, or the blue, or in any arbitrary combination in these. Moreover, there may be RGB colour channels that are the same at either side of the transition. These do not contribute to the distinctiveness. The “veil” might conceivably decrease distinctiveness. Finally, when transitions are present in several RGB colour channels, their polarity may possibly play a role. Thus, the structure of chromatic transitions is involved. An extensive discussion is offered in Appendix B.

From the overview of all data in Appendix C, one may glean various types of systematic variations due to the various parameters mentioned earlier. However, it is hard to obtain a coherent overall picture, so it is perhaps more useful to plot partial data in a more intuitive format.

We refer to the case of the transitions in only a single RGB colour channel as analogous colour pairs, of those in two of the RGB colour channels as incongruent colour pairs and of these in all of the three RGB channels as complementary pairs.

**Figure 16.**
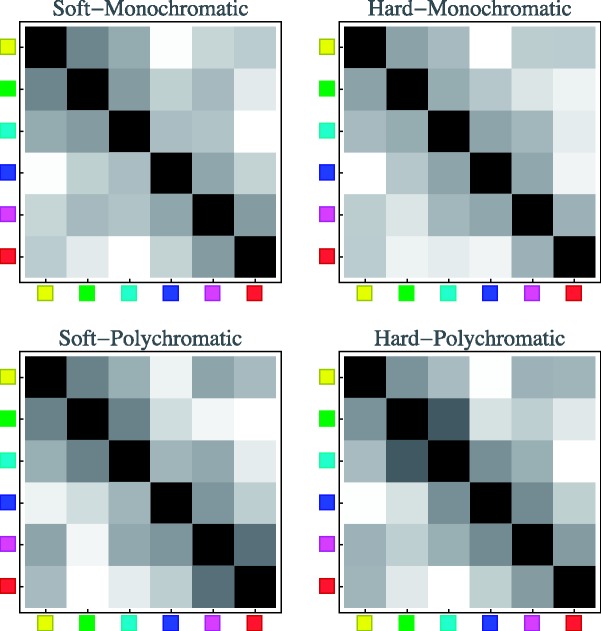
Array plots of the observations. The response range has been mapped on the full grey scale, where black represents zero and white represents the maximum response. Notice that the diagonal entries (self–self comparisons) are trivially zero.

The array plots presented in Figure 16 allow a quick overview of the data. Notice that the array plots appear to be roughly structured in bands running parallel to the main diagonal (which contains identical zeroes because representing comparisons of mutually identical patches). The plots are trivially symmetrical about the main diagonal, but the full plot helps visualise the data structure. (Notice that these arrays are double periodic!)

The various stimulus conditions appear to have only minor influence. In most cases, the distinctiveness of complementary pairs (CR, MG, or YB) is larger than the distinctiveness of analogous colour pairs (YG, GC, YG, CB, BM, MR, or RY), whereas the incoherent colour pair transitions appear to be in between. Thus, it makes sense to study the cases of analogous colour pairs and complementary colour pairs in more detail. Indeed, it is helpful to consider some special types of transitions that occur frequently in art and design (Chevreul, 1860; Küppers, 1976; Liebmann, 1927; Quiller, 1989; von Bezold, 1874; von Goethe, 1810).

**Figure 17.**
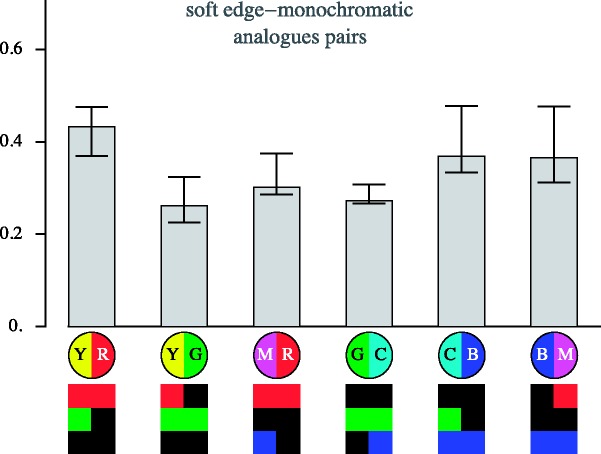
Overview of the results for pairs of the analogous colours for the case of the soft-edge, monochromatic stimuli. The bars show quartile values of the distinctiveness settings for all observers. Below are schematic representations of the nature of the transition (YR, YG, MR, GC, CB, and BM) in bipartite disks, and below that an analysis in terms of the individual RGB channels is given.

**Figure 18.**
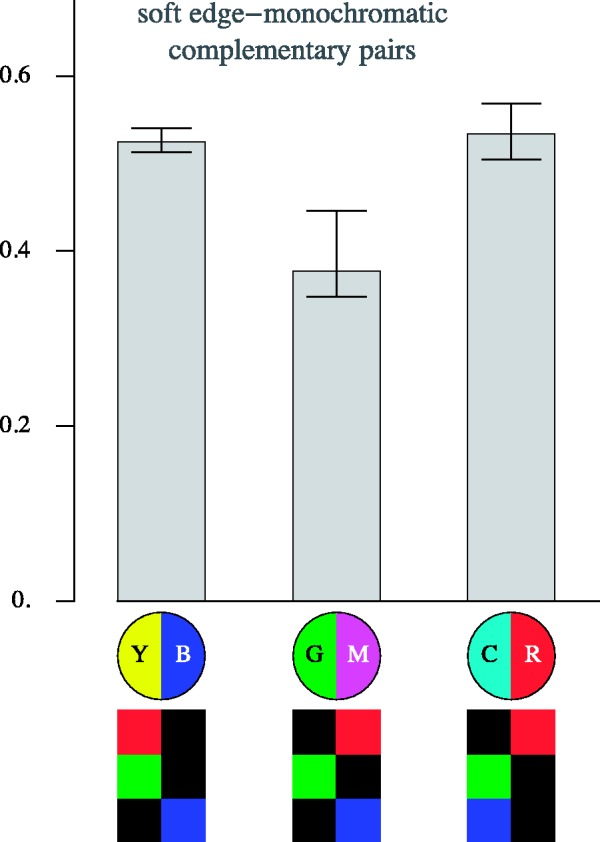
Results for the complementary colour pairs for the case of the soft-edge, monochromatic stimuli. The bars show quartile values of the distinctiveness settings for all observers. Below are schematic representations of the nature of the transition (YB, GM, and CR) in bipartite disks, and below that an analysis in terms of the individual RGB channels is given.

Figures 17 and 18 show more specific information about transitions involving analogous colour pairs and those involving complementary colour pairs. Apparently, transitions due to complementary pairs are more distinctive than those based on analogous colour pairs. (The interquartile ranges do not overlap, quartiles for the analogous pairs [0.29,0.32,0.40], for the complementary pairs [0.43,0.52,0.54].)

In the case of the transitions due to complementary colour pairs, one focusses on simultaneous differences in all RGB colour channels. It is the nature of the relative polarities of the transitions in the individual RGB colour channels that is relevant here. The GM transitions appear to be less distinct than either the YB or RC one.

In the case of transitions due to analogous colour pairs, one notices that a step in the green RGB colour channel appears to contribute relatively much to distinctiveness. Blue veils appear to less decrease in distinctiveness than either red or green veils.

These examples are only for the case of the soft-edge, monochromatic gamut case. However, from the full data set (Appendix B), one gleans that the specific cases are mutually only little different.

As a preliminary conclusion, chromatic transitions are definitely effective in forming visually effective transitions for all combinations of colour pairs. The distinctiveness differs relatively little with the various parameters, although there exist certainly systematic variations. In the Discussion section, we confront such findings with the general theoretical approaches mentioned earlier.

## Discussion

In a discussion of the empirical findings, it is of some interest to have a few prior expectations in mind. We discuss these first, before entering into a discussion of the data proper.

One major notion is no doubt the fact that the luminances of the cardinal colours are mutually quite different. From the data given in the Methods section, one gleans that the CIE luminances of the RGB are in the ratio 69:197:53, implying the following ratios for the six cardinal colours (see Appendix A):

**Table:** 

Y	100
G	74
C	94
B	20
M	46
R	26

Thus, one has a range of a factor of five, implying luminance contrasts up to almost 70%. One would certainly expect to see this reflected in the observations.

On the other hand, as we have shown in previous work, CIE luminance contrast is often of little value in predictions of Gestalt properties of images (Koenderink et al., 2017a, 2017b). Here, a maximum rule fares *much* better. The strength of a colour according to the maximum rule is proportional to its maximum RGB coordinate, thus is the same for all cardinal colours.

In the extreme case, this implies that blue (B = {0,0,1}) is of equal strength as yellow (Y = {1,1,0}); in both cases, the maximum coordinate is one. Yet, yellow is 5 times as luminous as blue! On the other hand, blue manages to counterbalance yellow, for taken together they make white ({0,0,1} + {1,1,0} = {1,1,1}).

In an equiluminant display of maximum brightness, one would set blue to its maximum intensity but would have to attenuate yellow by a factor of five. (When trying this on an electronic display, one should make sure to take the gamma into account.) Such a “yellow” actually looks dark brown. In a display where blue and yellow balance each other, in the sense of the painter's rules of composition, yellow and blue are both used in their full strengths.

These issues evidently play a role in the present case.

### Some A Priori Expectations

**Figure 19.**
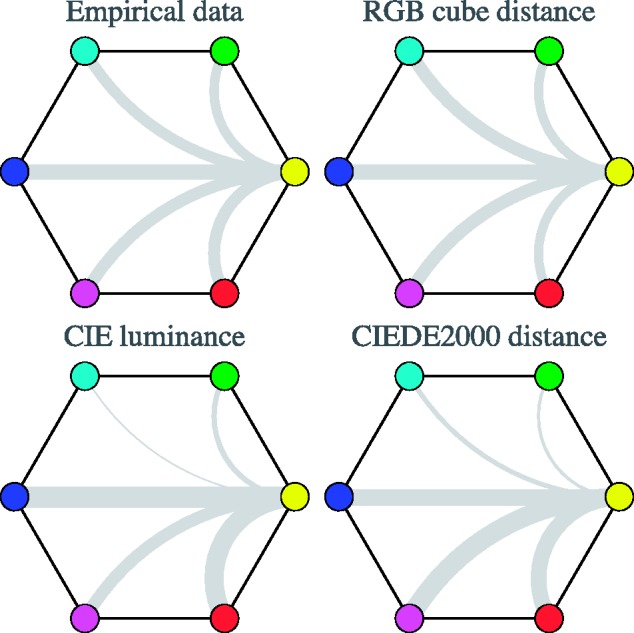
At top-left, an attempt at an overview of some data for the case of monochromatic, soft-edge stimuli (results for the other cases are quite similar). The hexagon represents the colour circle from the perspective of a fiducial cardinal colour, here yellow. The connections to each of the other cardinal colours are drawn in a thickness proportional to the distinctiveness of the corresponding bipartition. To present all data for a case, one needs to draw five more of this type of plots, but the case of a yellow fiducial is quite instructive by itself. The three other hexagons show various model predictions: raw RGB cube distance (top-right), CIE luminance (bottom-left), and CIEDE2000 distance (bottom-right). (Notice that the average thickness has been normalised to the same value in all cases.)

In Figure 19, bottom-left, we show expected contrast for some colour pairs on the hypothesis that CIE luminance contrast rules, the empirical data are shown in the same figure at top-left.

This is an alternative way to visualise the data. Such a representation lets one immediately notice the distinctiveness of transitions defined by two points on the colour circle. Depicting this for all data would involve several figures. However, the single example already suffices here. Even a cursory comparison with the observational data (see Figure 19) reveals that the CIE luminance contrast is a bad predictor for transition distinctiveness.

Apparently, the CIE luminance function does not fare well as an explicans of the data. However, one notices that although CIE luminance does a bad job in quantitative prediction, it gets at least some qualitative trends right. For instance, here, it picks out the YB transition as particularly distinctive, which is indeed the case at first sight, although far less outspoken in the empirical data. That luminance is not too important is already evident from the observation that it fails miserably as a tool to render a divisionist chromatic painting in grey tones, as demonstrated in Figure 1.

Does the maximum rule fare much better? Well, it is not that easy to use that for predictions; the most reasonable prediction would probably be to predict that the distinctiveness would not depend on the particular colour pair at all, although, perhaps, on the number of transitions in the RGB colour channels. Now this is indeed a prediction that fits the observational data much better (in a quantitative sense) than CIE luminance contrast (Figure 19, top-right), but it is perhaps not fully satisfactory because it loses sight of the (albeit minor) systematic variations that certainly exist.

To obtain a quantitative prediction, there evidently has to be a differentiation between R, G, and B colour channels. Because of this, it is of some interest to consider the nature of an RGB colour transition in some detail. (Appendix B gives additional information.)

For instance, consider the analogous colour pair yellow–green (YG). It can be understood (see Figure 15, left) as a red–black (RK) transition with a green veil because (YG = GG ∪ RK). Likewise, the colour pair magenta–blue (MB) can be understood as a red–black (RK) contrast with a blue veil, for (MB = BB ∪ RK). Thus, YG and MB are really the same transitions as seen in the red colour channel, but with different veils, namely green in the case of YG transition, or blue in the case of the MB transition.

What does this imply for the distinctiveness? Well, it might depend both upon the nature of the transition and on the influence of the veil. Similar reasoning applies to the other cases. The possibilities are limited through the fact that we did not admit white and black as colours of the left or right patches. However, there still remain many possibilities, so one is faced with something like a combinatorial explosion. Appendix B gives details. These are indeed necessary to parse the structure in the full data set provided in Appendix C.

We do not discuss the various systematic effects in full detail, but we notice that there exist effects of the nature of the colour channel transitions (that is, RK, GK, or BK as the case may be), the number of simultaneous transitions in different colour channels and their relative transition polarity (such as YB = RK ∪ GK ∪ KB vs. RC = RK ∪ KG ∪ KB, which differ in the polarity of the transition in the green colour channel when compared with that in the red and blue colour channels).

A model that would treat all RGB colour channel transitions and veils as potentially distinct would involve at least a dozen free parameters. We consider it far too detailed given the apparently minor influence of the various parameters relative to the overall effect, which is simply that the transition distinctiveness varies only little, thus may in the coarsest approximation be considered independent of the type of chromatic transition. However, it makes sense to attempt a much simplified description, taking only the number of transitions in the individual RGB colour channels and the presence or absence of veils into account. A fit to all data yields

$$D=0.406+0.025N_{t}-0.083N_{v},$$

where *D* is the distinctiveness, *N*_*t*_ the number of transitions in the individual RGB colour channels, and *N*_*v*_ the number of veils. The median error is 0.017, the maximum error 0.077. In view of the fact that the median of the interquartile ranges of the data for specific transitions is 0.055, the maximum 0.090, the simple description essentially explains the data quite well. Yet, it is only a phenomenological fit, and it does not take the type of the transitions or the colour of the veil into account, factors that on inspection certainly have an influence.

As taking all parameters into account seems indeed akin to overkill, we decided to confront the data with just the following three mutually categorically different models:

○ Luminance contrast is an obvious candidate for the prediction of edge contrast (Boynton & Kaiser, 1968; CIE, 1932; Smith & Guild, 1931–1932) and thus possibly chromatic transition distinctiveness. It would probably come out as the winner when vision scientists had to bet. As noted earlier, expectations cannot be high. However, any contending model should certainly be compared with this obvious candidate.
○ CIEDE2000 colour difference might well be the first bet of colour scientists (Alman, 1993; CIE, 2004; Luo et al., 2001; Moroney et al., 2002; Sharma, Wu, & Dalal, 2005). The CIEDE2000 distance is nonnegative and symmetric, but violates the triangle inequality, thus is not a “distance” in the formal sense. For instance, the CIEDE2000 distance for the detour green–cyan–magenta is less than the CIEDE2000 distance for the direct route green–magenta. About 3.2% of the random triangles of RGB colours violate the inequality of the triangle. As one shifts focus from tonal to chromatic palettes, this model should certainly be considered though.
○ as a poor man's alternative (Koenderink, 2010; Koenderink et al., 2017a, 2017b; Küppers, 1976), we propose Euclidean distance (Deza & Deza, 2009) in the RGB colour cube, where the RGB are raw coordinates in a gamma 2.2 linearised display. (For the set of cardinal colours that implies that the mutual distances are either 0, 1, $\sqrt{2}\approx 1.4$, or $\sqrt{3}\approx 1.7$.) That would be the obvious choice of the graphics programmer coding for generic displays, thinking colour specifications in terms of 3-bytes (or, combined, a 24-bit unsigned integer specified as hexadecimal). Modern artists and designers using computer graphics instead of chemical paints would most likely hold similar notions. After all, displays have evolved to fit human perception in circumstances of generic use.

Neither of these models does take the transition structure explicitly into account, although it is very well possible that they will implicitly depend upon transition type, number of colour channel transitions, veil, or relative transition polarity. For instance, in the case of the RGB cube model, the distance indeed increases monotonically with the number of colour channel transitions. Not surprisingly, the predictions of the three proposals are mutually very significantly correlated; see Table 2.

**Table 2.** Pairwise Correlations of the Three Proposed Distance Measures.

	Kendall's tau	Pearson's *r*
CIE luminance contrast–CIEDE2000 colour distance	.49	.73
CIE luminance contrast–RGB cube distance	.40	.59
CIEDE2000 colour distance–RGB cube distance	.67	.78

Thus, a comparison boils down to a detailed quantitative confrontation with the actual observations. Such a correlation is shown in a later section (Figure 23).

### Comparison of Results With Expectations

**Figure 20.**
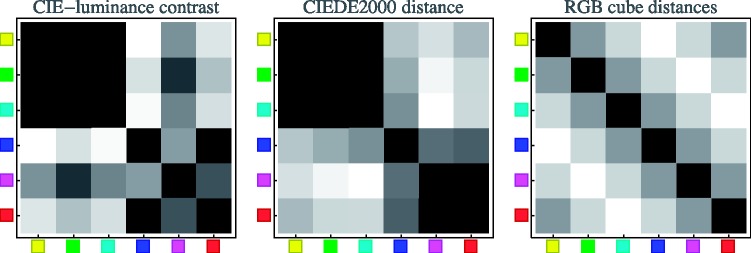
The array plots for the three models. Again, these have been normalised to use the full grey scale, from black (representing zero) to white (representing the maximum value). Compare Figure 16, representing the data. Notice again that the diagonal entries are trivially black and merely serve as a convenient landmark in comparing the patterns.

In Figure 20, we plot the predictions of the three models as array plots, allowing immediate comparison with the observations as presented in Figure 16. A cursory view reveals that the CIE luminance contrast and the CIEDE2000 colour distance predictions fail to capture the overall structure of the data, whereas the RGB cube model appears to do a more creditable job. A more detailed comparison is discussed in the next section.

**Figure 21.**
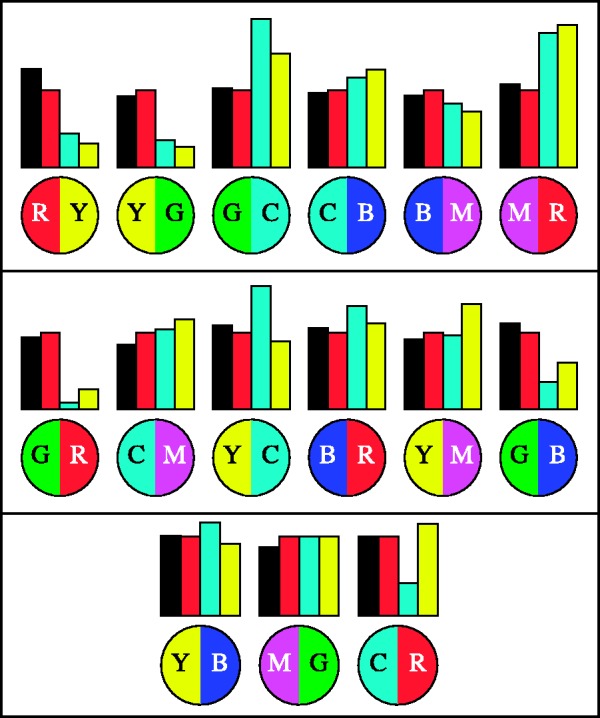
The median data for the hard-edge, monochromatic gamut case (black bars) compared with the predictions of the RGB cube colour distances (red bars), CIE luminance contrast (cyan bars), and CIE colour distance (yellow bars). We show the results for the analogous pairs in the top box, the results for the transitions with two transitions in the individual RGB channels in the centre box, and the results for the complementary pairs in the bottom box. To make a fair comparison possible, we normalised with respect to the median over each of the families analogous, incongruous, and complementary colour pairs.

In Figure 21, we present the predictions for the analogous colour pairs, the incongruent colour pairs and for the complementary colour pairs for all three models, together with the data. This figure shows the case of the hard-edge, monochromatic gamut. A single case is indeed sufficient because the results are so clear. (The other cases yield very similar distributions.)

Although none of the models predicts the observations in full detail, it is clear that the prediction from CIE luminance contrast is worst, that from the CIEDE2000 colour distance metric perhaps somewhat better, whereas the RGB cube distances predictions—although by no means perfect—at least manage to capture most of the structure of the data. More especially, the latter seem to do some justice to the qualitative structure of the observations.

This is especially evident from a study of the array plots (Figures 16 and 20).

The first, second, and third secondary diagonals in the array plots correspond to one, two, and three index-step differences along the colour circle on the (natural) 6-point scale, Y = 0, G = 1, C = 2, B = 3, M = 4, R = 5 (Bouma, 1946; Koenderink, 2010; Ostwald, 1917). They also equal the number of simultaneous transitions in the RGB colour channels and the distances 0, 1, $\sqrt{2}$, and $\sqrt{3}$ in the RGB cube. (Remember the doubly periodic structure of these matrices in checking these claims!)

Notice that the matrices for the cases of CIE luminance contrast or CIEDE2000 colour distance (Figure 20, left and centre) are quite differently structured from that of the RGB cube model (Figure 20, right). Both have a pronounced block structure and horizontal and vertical bands, mainly due to the special nature of blue. The structures of the response data matrices (Figure 16) are, of course, a bit noisy, but apparently fit the predictions of the simple RGB cube model more easily than either the CIE luminance contrast or the CIE colour distances. This is borne out by a correlation study shown in the following (Figure 23).

The case of a single transition in the RGB colour channels (the analogous colour pairs; Figure 21, top box) is perhaps of most analytic relevance. Both CIE luminance contrast and CIEDE2000 colour distance predict high distinguishability for the green–cyan and magenta–red transitions and extremely low distinguishability for the red–yellow and yellow–green transitions. In contradistinction, the RGB cube distances prediction has all transitions ex equo, as indeed (in view of the empirical spread) the data.

This major qualitative difference is reminiscent of the results we found earlier for chromatically defined Gestalt configurations (Koenderink et al., 2017a), the maximum rule had a much stronger predictive power than CIE luminance. Phenomenologically blue is related to black and yellow to white, a notion originally due to Goethe (von Goethe, 1810). This is indeed reflected in the CIE luminance (although Goethe and the CIE make strange bedfellows), for yellow has 83% of the luminance of white, but blue only 17% (in the case of our display unit, the conventional rule of thumb gives 90% and 10%). Yet, when added, blue cancels out the yellow, for the YB mixture is white, thus hueless. Thus, the blue has a colour-weight that far outweighs its luminance but is compatible with its equal brightness to yellow according to the maximum rule.

The case of the incongruent colour pairs yields similar conclusions (Figure 21, centre box). Both the CIE luminance and the CIEDE2000 distances get some prediction fully wrong. The predictions for the green–red and green–blue transitions are far too low. Only the RGB cube model does predict the data reasonably well.

The case of the complementary colour pairs is also of much interest (see Figure 21, bottom box). Here, only the CIE luminance contrast prediction does a bad job in case of the cyan–red transition; on the whole, all the other models do a creditable job of accounting for the data.

### Obvious Incompleteness of the Models

All three models considered here share the limitation that they base their predictions on the average colours of the patches at either side of the transition. Simply make the averages equal and all three models will declare the transition to be invisible to the human observer. Empirically, this is definitely not the case (te Pas & Koenderink, 2004).

**Figure 22.**
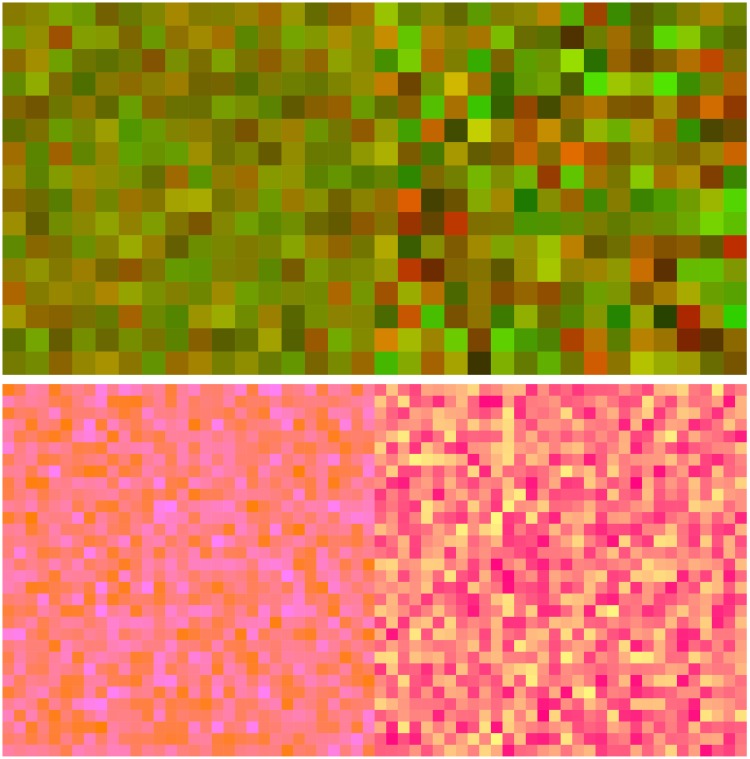
The hard-edge, polychromatic transition at top has zero distinctiveness in the prediction of all three models. Yet, we find it easy enough to see the distinction between the left- and right-hand areas, although the edge appears rather soft. In the example at bottom (likewise a polychromatic transition of zero distinctiveness in the prediction of all three models), one sees a sharp transition. Apparently, “zero distinctiveness” transitions come in different varieties and are a worthy target for detailed investigation.

Consider a case where one side will be a texture composed of fifty-fifty white or black checks and the other side a flat grey patch of matching luminance. Will you spot the transition? Obviously, big time! So this is a simple way to prove all three models wrong, or—perhaps more reasonably—*incomplete*. It is trivial to construct examples in the chromatic domain too; Figure 22 presents such examples.

**Figure 23.**
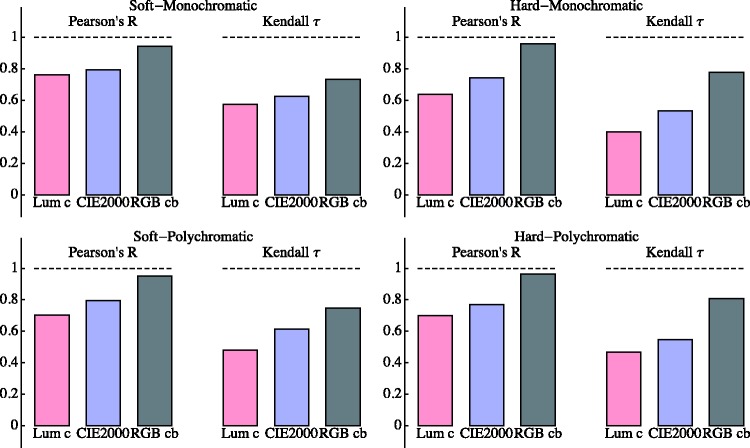
Correlations of the predictions of the three models with the observer settings.

It would be most interesting (te Pas & Koenderink, 2004) to have data from actual pointillist or divisionist paintings. Do artists intentionally use such “zero distinctiveness” transitions? Unfortunately, such data are not forthcoming. The reproductions found on the Internet are insufficiently controlled so as to be able to trust (necessarily) smallish samples. One would have to visit museums (and—in this case—numerous private collectors) with radiometric equipment to collect such data.

A more complete (we do not believe in ideals) model should take the dither statistics (for want of a better term) into account. Any model based on colorimetric considerations of patch averages (essentially “flat colours”) is necessarily flawed, or at least incomplete.

### Influence of the Stimulus Categories

What about the influence of the type of transition (hard or sharp edge) or the nature of the colour gamuts (monochromatic vs. polychromatic)? Not much is known about this in the cases of chromatically textured patches (Hansen et al., 2008; te Pas & Koenderink, 2004). It is evident that the transitions of polychromatic regions are less distinct and give rise to more uncertainty, than the corresponding monochromatic cases (Figure 10).

Perhaps surprisingly, empirically it makes very little difference whether the edges are hard or soft. That the difference in the structure of the transition (the artist's “edge quality”; Koenderink et al., 2016) is only minor might be taken as an indication that the comparison of regions is certainly not less important than the nature of the transition between them. Apparently, observers take account of the nature of regions rather than relying on the nature of the transitions. This implies that pointillist painting implies painting by macchie, unless the transitions are given special treatment, such as something chromatically akin to the Craik–O'Brien–Cornsweet illusion (Cornsweet, 1970). Examples of that are easy to find. The topic of pointillist chromatic macchie and their transitions still has many aspects that would yield interesting study material.

Of course, “edge quality” has various other uses than merely distinguishing regions; for instance, they are a primary mean in suggesting pictorial depth and relief.

## Conclusions

So what to conclude? We have collected some relevant data pertaining to pointillist–divisionist edges, but is there any insight to be gained from the perspective of the sciences? To be perfectly honest, our answer would have to be “little.” But anyway, this study provides some novel data and a balanced confrontation of the observations with generic expectations. The quantitative verdict from basic statistics is plotted in Figure 23. The rank correlation (Kendall's tau) and the linear product moment correlation (Pearson's *r*) coefficients evidently reflect each other. Apparently, the qualitative and quantitative perspectives on the data are not that different. One reading is that all models capture at least *some* of the message encoded in the data. Thus, the interest is in the details.

The main message is that CIE luminance contrast is not a great predictor of the distinctiveness of chromatic pointillist transitions. That is hardly a surprise because the painters were intentionally moving away from tonal contrasts. Apparently, they succeeded in that. However, the fact that they did and especially the way they achieved their aim remains perhaps still somewhat surprising from the perspective of generic vision science. Luminance contrast evidently does not figure. This is something we already encountered in an earlier study (Koenderink et al., 2017a).

Although the CIEDE2000 metric does evidently better than the predictions from luminance contrast, its performance falls perhaps short of expectations. In view of the rather extreme complexity of this metric (just compare it with the simple expression fitted earlier, the CIEDE2000 metric takes half a page!), should one not expect a much better fit to the data? Indeed, you may very well think so, but—as serious scientists—we are hardly in a position to comment.

Perhaps surprisingly, the “poor man's model,” Euclidean distance in the RGB cube as implemented on generic displays, uncorrected for its gamma of 2.2 apparently does fine. How can that be? Should not one correct for the nonlinear gamma transfer, convert to colorimetric coordinates (say CIE XYZ 1962), map to (a very nonlinear transformation) ${\text{L}}^{⋆}{\text{a}}^{⋆}{\text{b}}^{⋆}$-space, and use the Euclidean metric in *that* domain (the CIE1976 metric) perhaps subject to additional corrections and adjustments? Of course, one should! At least officially (CIE, 1932, 2004; Foley et al., 1990; Moroney et al., 2002; Poynton, 1993; Sharma et al., 2005; Smith & Guild, 1931–1932).

Here is our perspective on why the RGB cube model works so well, whereas it is obviously nonsensical, perhaps even in doubtful taste, from the perspective of the colour scientist: Manufacturers of display units are primarily interested in showing profit to their shareholders rather than the pursuit of science. They maximise their profits if their display units sell better than those of their immediate competitors. Their units will sell better (prices being similar) when they *look* better. Users expect the widest gamut, but even more importantly, they want red to look like red, green look like green, blue look like blue, red plus green look like yellow, green plus blue look as turquoise, blue plus red look as purple, and finally—but most importantly—red plus green plus blue look as white.

In the final analysis, they want their pictures to look good without much ado. Thus, they complain that the raw images from their electronic cameras (which are perfectly nice, linear spectroradiometric records) look so bland, much worse than the JPEGs (Foley et al., 1990) that are officially so much inferior to “raw.” That is to say, they (intuitively) want gamma two, or some kind of nonlinearity in that ballpark. Serious photography addicts spend lots of time and effort to convert their raw files to something close to what the camera's JPEG's renderer would give them by default. What they are doing by trial and error is finding a nonlinear transformation from the camera's so called correct, perfectly linear (currently about 3 × 14 bit) spectroradiometric data to some good looking (but only 3 × 8 bit) target. That is exactly what camera and display manufacturers are trying to offer the bulk (not willing to spend either time, or effort) of their customers by default. Recently, a trend in photography moves away from “raw developing” to “Straight-Out-Of-the-Camera” JPEGs. It only makes sense (Laing, 2017).

The upshot is that there is a strong evolutionary force for the generic RGB display to become intuitive, that is approximately uniform to the generic user. Little wonder then, that distances in such a “visually nice” (but physically very nonlinear) display space make very good visual sense; otherwise, the display units would simply fail to sell. It actually happens through engineering design being primarily driven by generic user demands rather than formal theory. What is perhaps surprising to the colour scientist is that distances realised this way manage to outperform the “official” measures.

The rank order of the RGB cube distances predictions comes perhaps surprisingly close to explaining our observations. It does fine in all cases, hard or soft edge, monochromatic or polychromatic. Yet, this model, if it deserves to be called a model at all, has essentially no background in colour science, except perhaps Goethe's (von Goethe, 1810) and his self-assigned pupil Schopenhauer's (Schopenhauer, 1816) ideas (Küppers colour cube model has a similar flavour; Küppers, 1976).

The major difference with either physiological models, based on receptor action spectra, or colorimetric models, based on empirical colour matching functions which are arbitrary linear combinations of the receptor action spectra, is that the Goethe–Schopenhauer models take “natural daylight” into account as a *causal factor*. In contradistinction, both physiological and colorimetric models regard the daylight spectrum as an essentially arbitrary, *external factor*. The fact that daylight looks achromatic is very relevant and indeed understandably so from an evolutionary perspective. The generic RGB system (towards which virtually all displays naturally evolve) can immediately be computed from the colour matching functions *and* the D65 spectrum, but not from the colour matching functions (nor the receptor action spectra) alone (Koenderink, 2010). Thus, the RGB system stands apart as the optimum representation for object colours under daylight illumination *given* the daylight spectrum and the colour matching functions (or action spectra).

Primitive and scientifically unmotivated as it may be, the trivial RGB cube distances model is very useful to the artist. It may explain that it is not overly hard to predict transition distinctiveness on the guts, by applying very simple, intuitive (usually preconscious) rules. It might be used in a variety of forms. For instance, substituting distance along the colour circle for distance in the RGB cube serves almost equally well. (Because the distance in the RGB cube varies monotonically with that along the colour circle for the set of cardinal colours, this is not surprising. Indeed, a numerical exercise reveals that the colour circle distance rule also beats CIEDE2000 colour distance predictions.) Distance along the colour circle may well capture the gist of an artist's precognitive notion of chromatic difference. Thus, a useful expectation of impact of chromatic transitions may not be that hard to acquire.

Consequently, these results lead to a number of very useful rules of thumb that have little or none relation to “official” colorimetry. For instance, purely chromatic contrasts lead to excellent visual partition of regions. They lead to clear distinctions whereas simultaneously leaving open various pictorial options. This gives the chromatic artist a head start over the tonal one. This alone (there exist a number of additional factors) already explains the reason for the widespread adoption of chromatic over tonal painting during the second half of the 19th century.

That luminance has only a minor influence on the present results has important consequences for various applications. Chromatic distinctions apparently depend primarily upon the separation of the participating hues along the locus of cardinal colours, a nonplanar hexagon lacking a begin or end point and thus implements the colour circle. Because the colour circle has the topology of S^1^, whereas tonal values have the topology of the linear segment II^1^, it is formally evident that there can exist no smooth, global map from chromatic contrasts to luminance contrasts.

**Figure 24.**
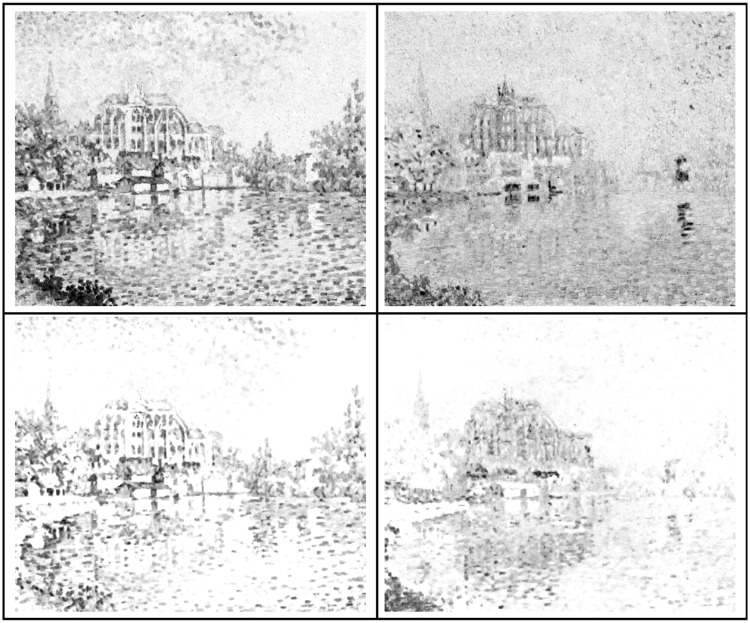
Some mutually very different, but equally valid grey-level interpretations of the Signac painting (Figure 1).

This implies that monochrome renderings of chromatically conceived paintings are necessarily compromises that may perhaps work in cases the chromatic structure of at least the major compositorial elements is limited—as it often is. In the latter case, an appropriate mapping from hues to tonal values may turn out to yield an acceptable monochrome rendering (examples shown in Figure 24). In many common cases, it is not hard to conceive of schemes that would yield a reasonable first shot at a monochrome rendering. For instance, consider the common analogous colours with complementary accent colour scheme (Quiller, 1989). This implies that the colours lie on a diameter of the colour wheel; thus, a map on a linear scale is immediate. The only choice is whether one would like to render the accents as light or dark. On the other hand, a rigid transformation based on luminance will work only as a random coincidence; there is absolutely no guarantee for success.

Of course, this is already well known to photographers. Programs such as Adobe's Photoshop (perhaps best known, though overkill for most users, explaining the plethora of simpler equivalents on the market) offer a variety of methods to transform a colour image to a monochrome one. Rarely will the experienced photographer be satisfied with any one of these though. More typically, an interpretation specific to the case will be sought. This is done via the “Channel Mixer Adjustment,” which has no formal structure, but is fully freestyle. Usually various distinct renderings might be considered suitable and some choice has to be made, based on aesthetic—not photometric—considerations.

**Figure 25.**
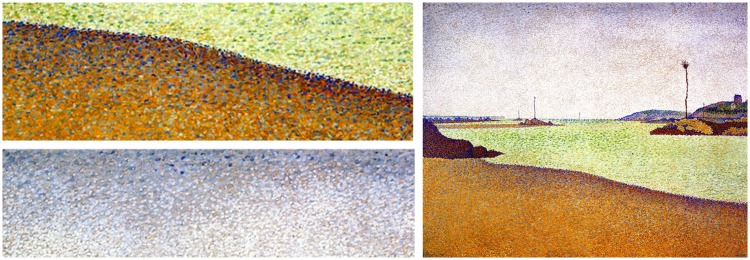
Paul Signac (1890), *The Beacons at Saint-Briac, Opus 210*, 65 × 81 cm, oil on canvas (public domain). At left, a hard on top and a soft edge below; both cutouts from the image at right. Notice the treatment of the hard edge; here, Signac modulated the edge in Craik–O'Brien–Cornsweet style.

Because black–white transitions are obviously powerful, there is more to distinctiveness than distance along the colour circle alone. The RGB cube distance model takes that into account, of course. So does the CIEDE2000 colour metric. (The luminance contrast model fails because it predicts equiluminant transitions to be invisible.) We have not really compared achromatic and chromatic transitions directly in this study. Moreover, we did not study various types of edge modulation, purely chromatic, or involving a luminance component (Figure 25). These are likely to be rewarding topics for further research.

Another loose end is the so called zero chromatic difference transitions. Such transitions will be visible through differences in spatial texture and statistics of chromatic dither. So their study borders on what vision research terms *texture discrimination* and *ensemble discrimination*, although they smoothly merge the type of problems considered here in a colour discrimination context. This is also a topic that is relatively unknown and invites closer study.

Apart from throwing some light on the technical side of Pointillism and Divisionism, the present work is of some interest to vision science proper. We show that in the case of “distemper,” that is of variegated and textured patterns, instead of flat colour, the artistic term for uniform, constant colour, the edges are of lesser importance than the regions separated by the edge.

*Polychromatic colour is seen by area rather than by edge*. Apparently, the impression of distinctiveness is mainly based on larger patches at either side of the transition (but see Hansen & Gegenfurtner, 2005). Moreover, perhaps surprisingly, such variegated colour can be treated colorimetrically in much the same way as flat colours, although they look far more appealing to the artistic eye.

## Figures and Tables

**Figure B1.**
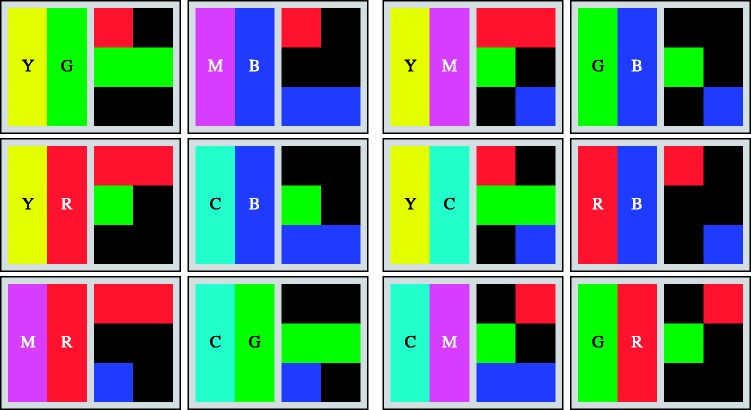
At left, the cases of a transition in a single RGB colour channel with veil in either one of the two remaining RGB colour channels. At right, the case of simultaneous edges in two RGB colour channels, with or without a veil in the third one.

**Figure B2.**
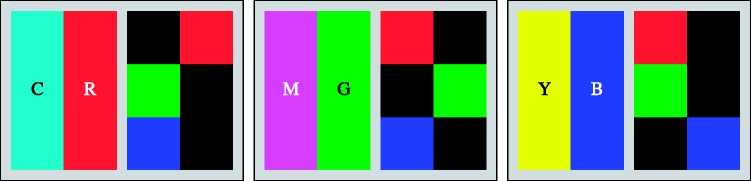
The case of simultaneous edges in all three RGB colour channels, in which case there will be no overlay. Here, the difference is in the polarity of the edges.

**Figure C1.**
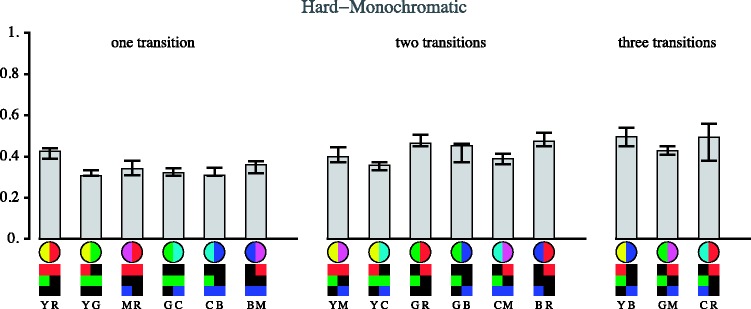
Normalised data for the *hard monochromatic* condition as quartiles over observers as bar plots for the various colour pairs. The transition structures are illustrated in Figures B1 and B2. The bars show quartile values of the distinctiveness settings for all observers.

**Figure C2.**
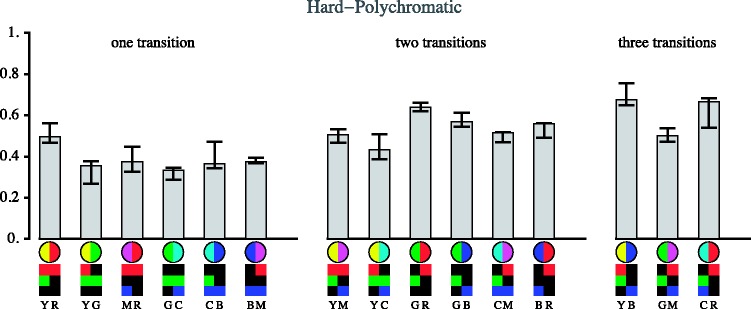
Normalised data for the *hard polychromatic* condition as quartiles over observers as bar plots for the various colour pairs. The transition structures are illustrated in Figures B1 and B2. The bars show quartile values of the distinctiveness settings for all observers.

**Figure C3.**
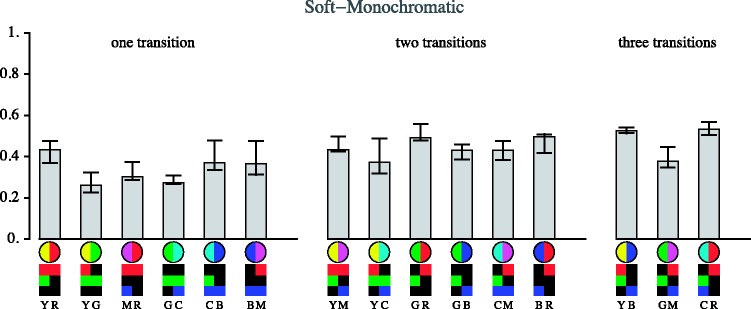
Normalised data for the *soft monochromatic* condition as quartiles over observers as bar plots for the various colour pairs. The transition structures are illustrated in Figures B1 and B2. The bars show quartile values of the distinctiveness settings for all observers.

**Figure C4.**
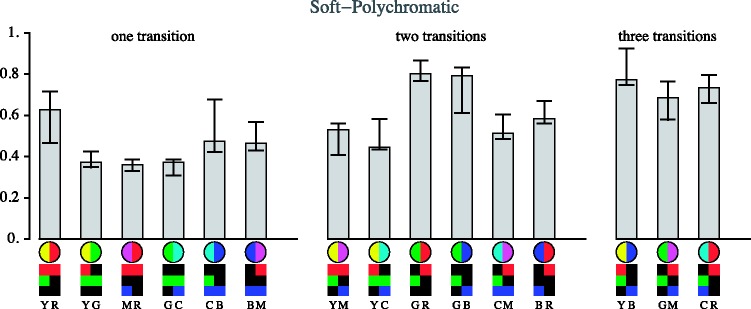
Normalised data for the *soft polychromatic* condition as quartiles over observers as bar plots for the various colour pairs. The transition structures are illustrated in Figures B1 and B2. The bars show quartile values of the distinctiveness settings for all observers.

## Notes

1. “Pointillism” refers to the spatial resolution of otherwise uniform areas into dots, whereas “Divisionism” refers to the use of a limited palette of discrete colours, without gradients obtained by mixture.

2. Here, we use “art” in the contemporary sense. Then, it is useful to distinguish (very roughly) between “illustration,” “design,” and “art.” These are all important, but mutually crucially different. Illustration and design can also be art, but need not be. Art often uses illustration or design, but need not, although it is very hard to skip design (in the guise of “composition”). In many cases, the same person might be illustrator, designer, or artist, depending on the current chore. This does in no way blur the distinctions though, the person would know. In this setting, “in the arts, only phenomenology counts” might be considered definitional.

## Appendix A. The RGB Representation

In this article, we use the RGB representation throughout. More extensive discussion can be found in Koenderink (2010) and Koenderink et al. (2017a, 2017b). Reasons are the immediate relation to basic colorimetry, combined with the close relation to phenomenology. Some relevant technical points are summarised here.

One disadvantage of basic colorimetry, say the CIE XYZ basis, is the arbitrariness in the choice of primaries and as a consequence the awkward match to phenomenology. Arbitrariness can simply be removed by moving to an invariant basis. This becomes possible after assuming a standard illuminant; we use CIE D65. It is a very common case, for instance, display devices are naturally limited by a maximum luminance achromatic colour (the display white).

In basic colorimetry, the only invariants are ratios of volumes; thus, one may define a unique basis by defining the primaries as the colours $\left\{C_{1},C_{2},C_{3}\right\}$ (say) such that $C_{1}+C_{2}+C_{3}=W$ (the colour of the illuminant), and C1^C2^C3 (the volume of the crate spanned by the primaries) is maximal. The solution is a spectral tripartition, with cut loci at 482.7 nm and 565.4 nm, defining a short wavelength part B, a medium wavelength part G, and a long wavelength part R. Any colour for a spectrum dominated by that of the illuminant is contained in the Schrödinger colour solid, whereas the crate R^G^B is the parallelepiped of largest volume inscribed in the colour solid. Writing P=rR+gG+bB defines the RGB colour coordinates for colour P. By construction, these coordinates are {1, 1, 1} for the colour of the illuminant W. One says that the {*r, g, b*} with 0≤r,g,b≤1 span the “RGB cube.”

This construction yields the optimum tricolour display, and indeed, all modern display units have closely converged to that. For many purposes (like this study), the actual display coordinates are close enough to the ideal RGB cube that the difference does not matter. (If so desired and the display unit has been spectrophotometrically calibrated [as here], one may transform to any desirable system because the RGB system is just a unique invariant representation of basic colorimetry.)

Any colour with RGB coordinates {*r, g, b*} can be written as kK+wW+cF, where K={0,0,0}, and F has one coordinate equal to 0, one coordinate equal to 1, and a third coordinate 0≤a≤1. Thus, any colour in the RGB cube is a linear interpolant between a primary cardinal colour (R = {1, 0, 0}, G = {0, 1, 0}, or B = {0, 0, 1}) and a secondary cardinal colour (C = {0, 1, 1}, M = {1, 0, 1}, or Y = {1, 1, 0}), mixed with white and black.

Here, the interpolations are between YG, GC, CB, BM, MR, or RY, implying a natural cyclical order YGCBMR. The connection with phenomenology is immediate: To most normal trichromatic observers, the sequence YGCBMR looks like yellow–green–cyan–blue–magenta–red, whereas KW looks like black and white. Here, one assumes a simple setting (say uniformly coloured disk on a grey background). That is why display units work in the expected way.

In the text, we use a “hue index” such that YGCBMR maps on the range [0…6), where 6 may be interpreted as 0 because of the periodicity. We use kK+wW+cF to define the “black content *k*,” the “white content *w*,” and the “colour content *c*,” the usual Ostwald-style parameters. Here, the connection with phenomenology is that observers readily estimate the black, white, and colour content in simple settings (say a uniformly coloured disk on a grey background).

## Appendix B. The Structure of Chromatic Transitions

The basic notions have already been introduced in the main text, especially Figure 15. A chromatic transition can be about any pair of cardinal colours, as we do not include either white or black in the possible patch colours. The analysis involves an analysis in terms of the transition in each of the RGB colour channels individually.

Notice that we consider the transitions PQ and QP (where P or Q stands for “any colour”) to be the same transition.

For each of these channels, there are three distinct cases to consider:

○ Both sides are black. In that case, we simply ignore this colour channel;
○ Both sides are identical, but not black. In that case, there is no transition in that channel, but there is something that might possibly influence the distinctness of the transition. We refer to this as a *veil*;
○ One side is black, and the other side is a primary colour. This is a *transition* in that colour channel. It has a certain polarity, specified by the presence of the colour in either the left- or the right-side patch. This is relevant only if there are transitions in all three colour channels, due to the fact that neither white nor black patches are allowed. We note which colour channel has a different polarity from the other two. It is this relative polarity that might be an effective factor.

The transition will be characterised by having one, two, or three transitions in the colour channels.

If there is one transition in some colour channel, there will be one other colour channel that contributes a veil (this follows from the constraint that patches are not allowed to be black or white). Thus, there are six distinct chromatic transitions of this type (Figure B1, left):

○ Transitions in the red channel allow YG (veil in the green colour channel) and MB (veil in the blue colour channel);
○ Transitions in the green channel allow CB (veil in the blue colour channel) and YR (veil in the red colour channel); and
○ Transitions in the blue channel allow MR (veil in the red colour channel) and CG (veil in the green colour channel).

Notice that these involve necessarily analogous colour pairs. Analog colour pairs are very frequently used in the arts, a very common choice being RY. The colours will be almost always used in strongly greyed down form, using the pure colours only sparingly (Quiller, 1989).

If there are transitions in two of the colour channels, there will be one other colour channel that might, or might not, contribute a veil (again, this follows from the constraint that patches are not allowed to be black or white). The transitions in the two colour channels are necessarily always of opposite polarity. Thus, there are again six distinct chromatic transitions of this type (Figure B1, right):

○ Transitions in the red and green colour channels allow MC (veil in the blue colour channel) and RG (no veil);
○ Transitions in the green and blue colour channels allow YM (veil in the red colour channel) and GB (no veil); and
○ Transitions in the blue and red colour channels allow CY (veil in the green colour channel) and BR (no veil).

Such transitions involve neither analogous nor complementary colour pairs. They involve either a pair of primary or a pair of secondary colours. These are combinations that are generally frowned upon in classical colour theory because these are considered not harmonious. You see them used in modern painting, especially RG, but they are not easy to handle and not generally appreciated. We refer to this family as “incongruent colour pairs” in the main text.

Finally, we consider the case that there are transitions in all three colour channels. The transitions involve necessarily a polarity contrast (once again, this follows from the constraint that patches are not allowed to be black or white). There are three mutually distinct cases (see Figure B2):

○ a cyan–red transition CR = KR ∪ GK ∪ BK,
○ a magenta–green transition MG = RK ∪ KG ∪ BK, and
○ a yellow–blue transition YB = RK ∪ GK ∪ KB.

(Here, the cup-operator  ∪  stands for the superposition of colour channels at both sides of the transition.) These transitions necessarily involve complementary colour pairs, often considered so called contrast colours. Such pairs work harmonious, but one has to be the dominant colour, the other used as accent. All combinations find use in the arts; the YB transition perhaps the most because it is read as “warm–cool” and is an effective tool in the creation of pictorial depth. One often sees landscape sketches in orange–turquoise blue, which even better aligns with the warm–cool direction (Albertazzi, Koenderink, & van Doorn, 2015). The transition MG (“sweet–sour”; Albertazzi et al., 2015) is orthogonal to this. It can be used effectively (Quiller, 1989) but more often occurs in more involved colour schemes.

## Appendix C. An Overview of All Data Ordered by Transition Type

In Figures C1 through C4, we present bar plots that represent all data in terms of quartiles. The various transitions have been grouped together on the basis of the number of transitions in the RGB colour channels. Thus, there are three such groups for each of the conditions—*soft monochromatic, hard monochromatic, soft polychromatic*, and *hard polychromatic*.

At left, one has the group of six analogous colour pairs; at right, the group of three complementary colour pairs at the right; and at the centre, the group of incongruent colour pairs.

In the group of analogous colour pairs, the major effect is due to which RGB colour channel has the transition, but the influence of the veil, although minor, is certainly significant in many cases. It appears that a blue veil has the least effect on distinctiveness, and a green veil has the most effect on distinctiveness.

In the group of six transitions involving incongruent pairs, one may have or not have a veil. This enables one to judge the influence of the presence of the veil (either there is a veil or there is none). The presence of the veil tends to decrease the distinctiveness of the transition, which is precisely what one would a priori expect.

In the group of three transitions with transitions in all colour channels, one sees that the yellow–blue (warm–cool) transition causes the most distinctive transitions, and the green–magenta (sour–sweet) causes the least distinctive transitions. The differences between these cases are (though generally significant) rather minor.
